# Development of tissue-engineered models of oral dysplasia and early invasive oral squamous cell carcinoma

**DOI:** 10.1038/bjc.2011.403

**Published:** 2011-10-11

**Authors:** H E Colley, V Hearnden, A V Jones, P H Weinreb, S M Violette, S MacNeil, M H Thornhill, C Murdoch

**Affiliations:** 1 Academic Unit of Oral and Maxillofacial Medicine and Surgery, School of Clinical Dentistry, University of Sheffield, Claremont Crescent, Sheffield S10 2TA, UK; 2 Department of Materials Science and Engineering, The Kroto Research Institute North Campus, University of Sheffield Broad Lane, Sheffield S3 7HQ, UK; 3 Academic Unit of Oral and Maxillofacial Pathology, School of Clinical Dentistry, University of Sheffield, Claremont Crescent, Sheffield S10 2TA, UK; 4 Biogen Idec Inc., 14 Cambridge Center, Cambridge, MA 02142, USA; 5 Stromedix Inc., 1 Canal Park, Suite 1120, Cambridge, MA 02141, USA

**Keywords:** squamous cell carcinoma, oral mucosa, dysplasia, invasion, tissue engineered

## Abstract

**Background:**

Current organotypic models of dysplasia and oral squamous cell carcinoma (OSCC) lack the complexity that mimics *in vivo* tissue. Here we describe a three-dimensional *in vitro* model of the oral epithelium that replicates tumour progression from dysplasia to an invasive phenotype.

**Methods:**

The OSCC cell lines were seeded as a cell suspension (D20, Cal27) or as multicellular tumour spheroids (FaDu) with oral fibroblasts on to a de-epidermised acellular dermis to generate tissue-engineered models and compared with patient biopsies.

**Results:**

The D20 and Cal27 cells generated a model of epithelial dysplasia. Overtime Cal27 cells traversed the basement membrane and invaded the connective tissue to reproduce features of early invasive OSCC. When seeded onto a model of the normal oral mucosa, FaDu spheroids produced a histological picture mimicking carcinoma *in situ* with severe cellular atypia juxtaposed to normal epithelium.

**Conclusion:**

It is possible to culture *in vitro* models with the morphological appearance and histological characteristics of dysplasia and tumour cell invasion seen *in vivo* using native dermis. Such models could facilitate study of the molecular processes involved in malignant transformation, invasion and tumour growth as well as *in vitro* testing of new treatments, diagnostic tests and drug delivery systems for OSCC.

Head and neck squamous cell carcinoma (HNSCC), which includes oral squamous cell carcinoma (OSCC), is the sixth most common malignancy worldwide (Argiris *et al*, 2008), and despite improved patient outcomes in a range of cancers the prognostic implications of HNSCC remain poor. With no established screening methods, visual identification of oral cancers is imperative for early detection, although recent developments of aneuploidy analysis for premalignant lesions may help to predict those lesions at risk of developing malignancy (Torres-Rendon *et al*, 2009; Bradley *et al*, 2010). Premalignant lesions have a variety of clinical presentations and may present as leukoplakia (white patch) or erythroplakia (red patch). Although erythroplakia is more likely to show histological features of dysplasias that then progress to OSCC, only a small proportion of all premalignant lesions progress to malignancy (Reibel, 2003).

Numerous aetiological factors are involved in the transformation of normal oral mucosa (NOM) to premalignant dysplastic lesions that may then develop into oral carcinoma (Jefferies and Foulkes, 2001; Leemans *et al*, 2011). Using molecular techniques to examine loss of heterozygosity, gene mutations and gene transcription patterns, investigators have shown that oral carcinogenesis is a progressive multistep process resulting from the clonal expansion of a cancer stem cell with a *TP53* mutation. Initially, a small patch of these mutated cells are present in the oral mucosa. These cells then acquire additional cancer-related genetic alterations and expand to form a larger field of abnormal cells that, with the acquisition of further genetic changes, then progress to invasive cancer (Califano *et al*, 1996, 2000; Braakhuis *et al*, 2003). In fact, current evidence suggests that oral premalignant lesions share genetic alterations and transcription profiles that are closer to the malignant lesions they go on to form than the transcription profiles of premalignant lesions that do not progress to cancer (Ha *et al*, 2003; Hunter *et al*, 2006). Thus, it seems that OSCC genetic signatures are already embedded in the premalignant cells that are destined to progress to malignancy.

Histological analysis of epithelial dysplasia is graded as mild, moderate or severe depending on the extent of atypia in the oral mucosa. Full-thickness dysplasia is classified as carcinoma *in situ* and only becomes an early invasive OSCC once tumour cells have penetrated through the basement membrane and infiltrated into the connective tissue. The clinical appearance of a dysplastic lesion is not a good guide to the level of dysplasia, and hence potential malignancy or malignancy can only be assessed by biopsy and histopathological evaluation. Despite recent therapeutic developments (Mao *et al*, 1998; Rudin *et al*, 2003; Rhee *et al*, 2004), there is currently no effective treatment for dysplasia but to reduce the associated risk factors for malignant change, such as smoking and alcohol consumption, and to monitor for malignant progression.

Although a valuable tool in oral cancer research, monolayer cultures of epithelial cells do not achieve the complexity of the oral mucosa *in vivo.* Important features innate to this organised tissue, such as cell–cell interactions, control of proliferation, differentiation, the deposition of a basement membrane and the controlling influence of the connective tissue (Moharamzadeh *et al*, 2007), are all lost in monolayer culture. Xenograft animal tumour models or tumours generated by epithelial transformation using chemical carcinogens have been successful in identifying important genes, molecules and mechanisms involved in oral cancer development and progression (Kim, 2009). Although several *in vivo* models for studying head and neck are available, none are fully satisfactory as they are often difficult to establish, produce varying results and raise important questions about the genetic differences between humans and animals, and whether xenograft or chemically induced tumour models are representative of human oral cancer (Prime *et al*, 1986; Kim, 2009).

Tissue-engineered three-dimensional (3D) models of the NOM are now well established for *in vitro* investigations (reviewed in Moharamzadeh *et al*, 2007) and have more recently been used for clinical applications (Bhargava *et al*, 2008). The keratinocyte organotypic co-culture model originally developed by Fusenig *et al* (1983) has been used by investigators to mimic oral dysplasia or cancer (Nystrom *et al*, 2005; Costea *et al*, 2006; Vigneswaran *et al*, 2006; Gaballah *et al*, 2008; Marsh *et al*, 2011). This method involves the culture of keratinocytes at an air-to-liquid interface on a fibroblast-containing collagen type I matrix. Interactions between the mesenchyme and epithelium are essential for normal epithelium development and maintenance. Crosstalk between the stroma and the overlying epithelium is known to influence a number of processes including keratinocyte proliferation, migration, differentiation and the formation of a basement membrane at the epithelial-dermal junction (EDJ) by synthesis of extracellular matrix (ECM) components (Saintigny *et al*, 1993). It is therefore not surprising that the stromal microenvironment is known to play a significant role in tumour invasion and progression (Mueller and Fusenig, 2004; Marsh *et al*, 2011). However, the basic homogeneous structure of the connective tissue (Wolf *et al*, 2009) and lack of a native basement membrane (Chung *et al*, 1997; Yadev *et al*, 2011) in collagen-based models allows only a simplistic representation of the native stromal component. Mucosal models that utilise a more complex, native, connective tissue containing a defined basement membrane to investigate oral dysplasia and invasive carcinoma may provide a greater insight into the molecular mechanisms controlling premalignant dysplasia and invasion *in vivo*.

The aim of this study was to develop and optimise a tissue-engineered, full-thickness model of squamous cell carcinoma of the oral mucosa from a dysplastic phenotype to early invasion using native human dermis. Such models could facilitate study of the mechanistic aspects of disease processes that are involved in malignant transformation, early invasion and tumour growth as well as facilitate final stage *in vitro* testing of new treatments, diagnostic tests and drug delivery systems for OSCC.

## Materials and methods

### Culture of HNSCC cell lines

This study used the following HNSCC cell lines: Cal27 (American Tissue Culture Collection (Manassas, VA, USA), CRL-2095) that was originally isolated from a 56-year-old Caucasian male with a squamous cell carcinoma of the tongue (Gioanni *et al*, 1988); FaDu (LGC Promochem, Middlesex, UK) that was isolated from a hypopharyngeal tumour (Rangan, 1972); DOK (ECACC, Health Protection Agency Culture Collections, Salisbury, UK), a dysplastic oral keratinocyte cell line that was originally isolated from the dorsal tongue of a 57-year-old male (Chang *et al*, 1992); and D20 that was derived from a lateral tongue dysplasia (McGregor *et al*, 1997). Cal27 and DOK cells were routinely cultured in Dulbecco's modified Eagle's medium (DMEM) and FaDu cells in RPMI-1640 supplemented with 10% (v/v) fetal calf serum (FCS; BioSera, East Sussex, UK), 2 mM L-Glutamine, 100 IU ml^–1^ penicillin and 100 *μ*g ml^–1^ streptomycin (Sigma, Poole, UK). Medium for the culture of DOK cells was also supplemented with 5 *μ*g ml^–1^ hydrocortisone. D20 cells were cultured in flavin- and adenine-enriched medium: DMEM and Ham's F12 medium in a 3 : 1 (v/v) ratio supplemented with 10% (v/v) FCS, 0.1 *μ*M cholera toxin, 10 ng ml^–1^ of epidermal growth factor (EGF), 0.4 *μ*g ml^–1^ hydrocortisone, 0.18 mM adenine, 5 *μ*g ml^–1^ insulin, 5 *μ*g ml^–1^ transferrin, 2 mM glutamine, 0.2 *μ*M triiodothyronine, 0.625 *μ*g ml^–1^ amphotericin B, 100 IU ml^–1^ penicillin and 100 *μ*g ml^–1^ streptomycin (Allen-Hoffmann and Rheinwald, 1984).

### Flow cytometry

Keratinocytes were removed from tissue culture flasks non-enzymatically, centrifuged at 250 g for 5 min and re-suspended at 1 × 10^7^ cells per ml in cold PBS supplemented with 0.1% BSA, 0.1% sodium azide (FACs buffer) and kept on ice. Cells (5 × 10^5^ cells) were incubated for 30 min on ice with 10 *μ*g ml^–1^ anti-*α*v*β*6 mouse monoclonal antibody (clone 6.3G9) or IgG isotype-matched control. Following washes with FACs buffer, cells were incubated with AlexaFlour 488-conjugated anti-mouse antibody (Invitrogen, Paisley, UK) for 30 min on ice in the dark, then washed twice and re-suspended with FACs buffer. Flow cytometry was performed using a FACsCalibur (Becton Dickinson, San Jose, CA, USA); propidium iodide was used to gate out nonviable cells and data were analysed using CellQuest Software (Becton Dickinson).

### Multicellular tumour spheroid (MCTS) production

Multicellular tumour spheroid were generated from FaDu cells using the liquid overlay method as previously described (Carlsson and Yuhas, 1984). Briefly, 100 *μ*l of FaDu cells (1.2 × 10^5^ per ml) were added to each well of a 96-well plate previously coated with 1.5% type V agarose (w/v in RPMI). The cells were incubated at 37 °C, 5% CO_2_ and monitored at 24 h for MCTS production. Medium was changed every 48 h by removing and re-placing 50% of the medium.

### Primary human oral keratinocyte and fibroblast isolation

Normal oral keratinocytes (NOKs) and fibroblasts (NOFs) were isolated from biopsies obtained from the buccal and gingival oral mucosa from patients during routine dental procedures with written, informed consent (ethical approval number 09/H1308/66) as previously described (Hearnden *et al*, 2009). Briefly, biopsies were incubated overnight at 4 °C in 0.1% w/v trypsin solution supplemented with 100 IU ml penicillin, 100 mg ml^–1^ streptomycin and 0.625 *μ*g ml^–1^ amphotericin B. Following enzymatic digestion, the epithelium was removed from the connective tissue and oral keratinocytes isolated by gently scraping the papillary surface of the epithelium and the uppermost side of the connective tissue layer. Keratinocytes were cultured on irradiated mouse 3T3 feeder layers in flavin- and adenine-enriched medium.

The NOFs were isolated from the connective tissue by fine mincing followed by digestion with 0.5% (w/v) collagenase A overnight at 37 °C. The NOFs were collected by centrifugation and then cultured in DMEM supplemented with 10% FCS, 2 mM glutamine, 100 IU ml^–1^ penicillin and 100 *μ*g ml^–1^ streptomycin.

### Production of de-epidermised acellular dermis (DED)

De-epidermised acellular dermis was produced from either split-thickness skin grafts or commercially available cadaver skin (Euroskin, Beverwijk, Holland) and processed as previously described (Eves *et al*, 2000). Split-thickness skin grafts were immersed in 1 M sodium chloride for 24 h at 37 °C to allow removal of the epidermis. The resultant DED samples were then washed extensively in PBS and stored at 4 °C in DMEM until use.

### Production of tissue-engineered oral mucosal models

**Figure 1:**
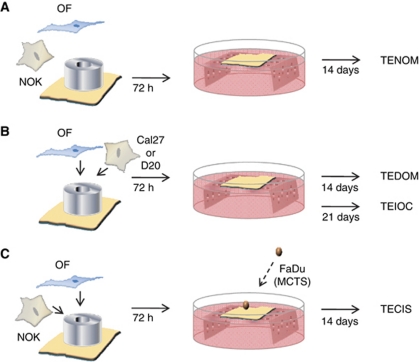
Schematic illustration showing the methodology for producing the full-thickness tissue-engineered oral mucosal models. To produce the tissue-engineered normal oral mucosa model (TENOM), normal oral fibroblasts (OFs) and normal oral keratinocytes (NOKs) were seeded onto a DED scaffold within a 0.8 cm^2^ steel ring. After 72 h, the composites were raised to an air/liquid interface and cultured for a further 14 days (**A**). To recreate an *in vitro* model of dysplastic oral mucosa (TEDOM) and early invasive oral carcinoma (TEIOC), the NOKs were replaced with OSCC cells (Cal27 or D20) and cultured for either 14 or 21 days, respectively (**B**). The addition of a MCTS to the TENOM produced a model closely mimicking carcinoma *in situ* (TECIS) (**C**).

De-epidermised acellular dermis was incubated in fresh DMEM for 48 h at 37 °C to confirm sterility. Then, 15 mm^2^ pieces were transferred to six-well plates (papillary surface uppermost) and an 8 mm diameter steel ring placed on top of each piece and immersed in medium. Tissue-engineered normal oral mucosa models (TENOM), (Figure 1A) were produced by seeding 5 × 10^5^ NOKs and 5 × 10^5^ NOFs in the centre of the steel ring. For the tissue-engineered dysplastic oral mucosa models (TEDOM, Figure 1B), either 2.5 × 10^5^ Cal27 or D20 cells and 5 × 10^5^ NOFs were used, and for the tissue-engineered early invasive oral carcinoma (TEIOC, Figure 1B) models, 2.5 × 10^5^ Cal27 cells and 5 × 10^5^ NOFs were used. Medium within the ring was replaced after 24 and 48 h. After 72 h, the ring was removed and the composites placed on to stainless steel grids with medium added to the underside of the composite model to allow culture at the air-to-liquid interface. To generate tissue-engineered carcinoma *in situ* (TECIS), (Figure 1C) models, 7-day-old FaDu MCTS were added to TENOM. For all models, medium was changed 2–3 times a week and the composites fixed at day 14 for TENOM, TEDOM, TECIS and at day 21 for TEIOC in 10% buffered formalin for 48 h. All models were then bisected and paraffin embedded.

### Immunohistochemistry

Paraffin-embedded tissue sections (5 *μ*m) were prepared from tissue-engineered mucosal models and for comparative purposes from paraffin-embedded blocks of NOM and archival HNSCC biopsies in accordance with the Sheffield Research Ethics Committee approval (Ref: 07/H1309/105). Sections were dewaxed, rehydrated and either stained with haematoxylin and eosin (H&E) or subjected to immunohistochemistry, in which case endogenous peroxidase was neutralised with 3% hydrogen peroxide for 20 min. Following antigen retrieval, sections were blocked using protein-free blocking solution (Dako, Copenhagen, Denmark) for 20 min at room temperature and then mouse monoclonal primary antibody (diluted in PBS) applied for 1 h at room temperature (see Table 1 for antibodies, dilutions and antigen retrieval method used). Secondary antibody and avidin-biotin complex (ABC) provided with Vectastain Elite ABC kit (Vector Labs, Peterborough, UK) were then used in accordance with the manufacturer's instructions. Finally, 3′-diaminobenzidine tetrahydrochloride (DAB) (Vector Labs) was used to visualise peroxidase activity and the sections were counterstained with haematoxylin, dehydrated and mounted in DPX. Images were taken using an Olympus BX51 microscope and Colour view IIIu camera with associated Cell^D software (Olympus Soft Imaging Solutions, GmbH, Münster, Germany).

## Results

### Full-thickness tissue-engineered normal oral mucosa model

**Figure 2:**
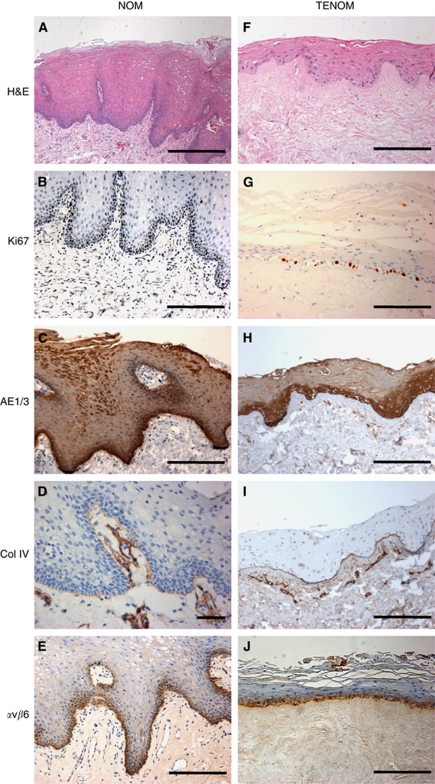
Representative sections of normal oral mucosa taken from patient biopsies (NOM) (**A**–**E**) and tissue-engineered normal oral mucosa constructs (TENOM) (**F**–**J**). TENOM models were generated by culturing normal oral keratinocytes and normal oral fibroblasts on DED at an air/liquid interface for 14 days. Sections have been stained with H&E (**A** and **F**) and immunohistochemically stained to show the pattern of expression of Ki67 (**B** and **G**), AE1/3 (**C** and **H**), collagen type IV (Col IV; **D** and **I**) and integrin *α*v*β*6 (**E** and **J**) and counterstained with haematoxylin. Scale bar indicates 200 *μ*m.

To generate a full-thickness TENOM, oral keratinocytes and oral fibroblasts were cultured on a DED scaffold at an air-to-liquid interface for 14 days. The oral keratinocytes migrated and proliferated laterally over the dermis as evident from cross-sectional histological analysis with the epithelia at the centre of the composite being thicker and tapering off towards the edges (data not shown). Histologically, the TENOM demonstrated normal architectural morphology, epithelial maturation and surface keratinisation, a convoluted EDJ and a fibroblast-populated dermis that closely replicates the histological appearance of NOM (Figure 2A and F). A similar proliferation index to NOM was evident upon Ki67 staining with proliferating cells predominately located in the basal or para-basal cell layer (Figure 2B and G). The pan-cytokeratin antibody anti-AE1/3 showed positive expression throughout the thickness of the TENOM epithelium with increased intensity in the lower third of the epithelium and was comparable to the NOM (Figure 2C and H). Immunohistochemical staining for collagen IV, a component of basement membranes, showed that TENOM forms a basement membrane at the junction of the epithelium and the underlying connective tissue, as well as around vascular spaces in the connective tissue, similar to that seen in NOM (Figure 2D and I). Finally, an antibody against integrin *α*v*β*6 (Weinreb *et al*, 2004) revealed positive staining associated with the keratinocytes in the basal cell layer of both NOM and TENOM (Figure 2E and J).

### Tissue-engineered dysplastic oral mucosa model

**Figure 3:**
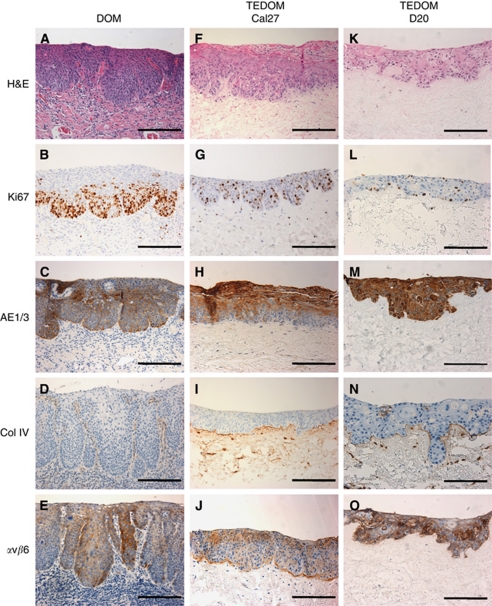
Representative sections of dysplastic oral mucosa taken from patient biopsies (DOM) (**A**–**E**) and tissue-engineered dysplastic oral mucosa constructs (TEDOM) (**F**–**J**). TEDOM models were generated by culturing D20 or Cal27 cells and normal oral fibroblasts on DED at an air-to-liquid interface for 14 days. Sections were stained with H&E (**A**, **F** and **K**) and immunohistochemically stained to show the pattern of expression of Ki67 (**B**, **G** and **L**), AE1/3 (**C**, **H** and **M**), collagen type IV (Col IV; **D**, **I** and **N**) and integrin *α*v*β*6 (**E**, **J** and **O**) and counterstained with haematoxylin. Scale bar indicates 200 *μ*m.

During the transformation from normal to premalignant lesions, normal proliferation and differentiation of the mucosal epithelium is disrupted, resulting in a dysplastic architecture. In dysplastic oral mucosa, the rete processes become rounded, drop-shaped and bulbous, and there is loss of normal keratinocyte maturation. To model these changes *in vitro*, NOKs used for the TENOM were replaced with the dysplastic cell line D20 or malignant Cal27 cells and again seeded with NOFs on to a DED scaffold. The TEDOM displays architectural and cytological changes typically associated with clinical lesions histologically classified as severely dysplastic. Similar to the clinical lesion, the TEDOM for both D20 and Cal27 cells show bulbous rete processes and abnormal keratinocyte maturation (Figure 3A, F and K). In addition, abnormal keratinocyte proliferation patterns are evident in the models and match that seen in the dysplastic biopsy with proliferating cells extending well into the supra-basal epithelial layers (Figure 3B, G and L). The ratio of ki67-positive cells to nonproliferating cells is similar in the biopsy compared with D20 and Cal27 models and there is an increased proliferation index of Ki67-positive epithelial cells along with abnormally high levels of mitotic figures (Figure 3B, G and L). The resultant loss of normal maturation and disrupted keratinocyte differentiation is also evidenced by AE1/3 staining (Figure 3C, H and M). Further cytokeratin profile staining showed positive expression of CK5/6 in both D20 and Cal27 models, which is characteristic of oral tumours, whereas staining for CK7 (characteristic of lung and ovarian tumours) or CK20 (characteristic of colonic or pancreatic tumours) was negative (data not shown). However, despite these histological changes, immunohistochemical staining for collagen type IV shows a well-defined, continuous basement membrane with no evidence of malignant invasion and clearly mimics the *in vivo* situation of dysplastic epithelium (Figure 3D, I and N). Expression of *α*v*β*6 is dramatically changed in DOM where it is expressed erratically throughout the entire epithelium as well as on some basal cells. A similar pattern of *α*v*β*6 expression was observed in the TEDOM of D20 and Cal27 models when compared with expression in DOM (Figure 3E, J and O). Similar results to those described above were also found using the dysplastic cell line DOK (Supplementary Figure 1).

### Tissue-engineered model of a severely dysplastic epithelium (carcinoma *in situ*) surrounded by normal oral mucosa

**Figure 4:**
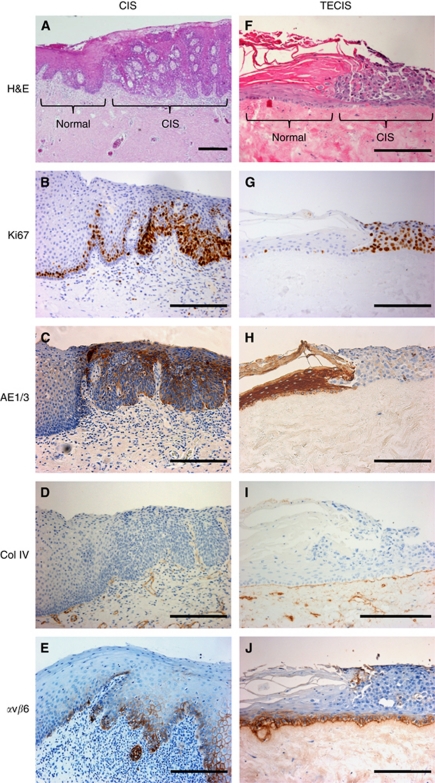
Representative sections of oral mucosa taken from patient biopsies pathohistologically classified as carcinoma *in situ* (CIS) (**A**–**E**) and a tissue-engineered model of carcinomas *in situ* (TECIS) (**F**–**J**). TECIS models were generated by the addition of a MCTS to the TENOM model and further culture at an air/liquid interface for 14 days. Sections have been stained with H&E (**A** and **F**) and immunohistochemically stained to show the pattern of expression of Ki67 (**B** and **G**), AE1/3 (**C** and **H**), collagen type IV (Coll IV; **D** and **I**) and integrin *α*v*β*6 (**E** and **J**) and counterstained with haematoxylin. Scale bar indicates 200 *μ*m. In each case, relatively normal tissue is seen to the left and CIS to the right of each section.

**Figure 5:**
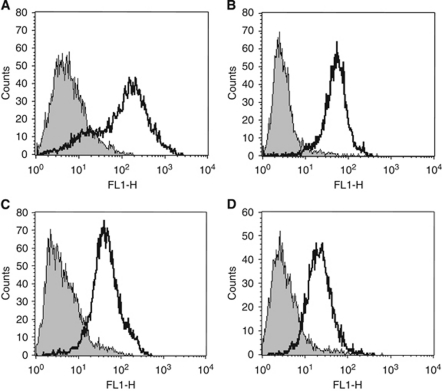
Flow cytometric analysis showing expression of cell surface *α*v*β*6 on monolayer cultures of normal oral keratinocytes (**A**), DOK (**B**), Cal27 (**C**) and FaDu (**D**). Histograms show expression of *α*v*β*6 (open histogram) compared with isotype-matched control IgG (grey-filled histogram). The *x* axis is log scale fluorescence.

Biopsies are routinely taken from the visual boundary of the oral lesion to allow histological examination of normal epithelium juxtaposed to diseased tissue. A tissue-engineered model containing both a dysplastic and normal element would therefore be valuable to enable examination of the interface between diseased and normal tissue in parallel. To model this, the OSCC FaDu cell line was cultured as a MCTS (to enable easy manipulation of the cells into the desired position; Cal27 cells do not produce satisfactory MCTS and hence were not used in this model) and incorporated within a TENOM. The model produced a histological picture closely resembling carcinoma *in situ* with a highly disorganised epithelial architecture showing severe cytological atypia involving the entire thickness of the epithelium and lying adjacent to an otherwise normal epithelium (Figure 4A and F). High proliferation indices with large numbers of Ki67-positive supra-basal keratinocytes for FaDu cells in the TECIS model and dysplastic cells in the oral biopsy were seen in contrast to the typical intermittently Ki67-positive polarised para-basal cells in the adjacent areas of TENOM and NOM (Figure 4B and G). In the TECIS model the FaDu cells showed reduced expression of keratins, staining weakly for the pan-cytokeratin marker AE1/3 in comparison with the adjacent ‘normal tissue’ that was stained throughout the epithelium. This was in contrast to the oral biopsy that showed disrupted AE1/3 cytokeratin staining throughout the dysplastic epithelium (Figure 4C and H). Comparable to the CIS biopsy, FaDu cells in the TECIS did not penetrate the basement membrane and invade into the underlying connective tissue, and a collagen IV-positive intact basement membrane was present in both cases (Figure 4D and I). Expression of *α*v*β*6 was weak in both CIS and TECIS and the expression was restricted to the basal epithelial cells (Figure 4E and J). Flow cytometric analysis showed that NOK, DOK, Cal27 and FaDu all express cell surface *α*v*β*6 when grown as monolayer cultures (Figure 5), suggesting that expression of this receptor dramatically changes when grown in 3D models and may be associated with a less well-differentiated phenotype.

### Tissue-engineered model of early invasive oral squamous cell carcinoma

**Figure 6:**
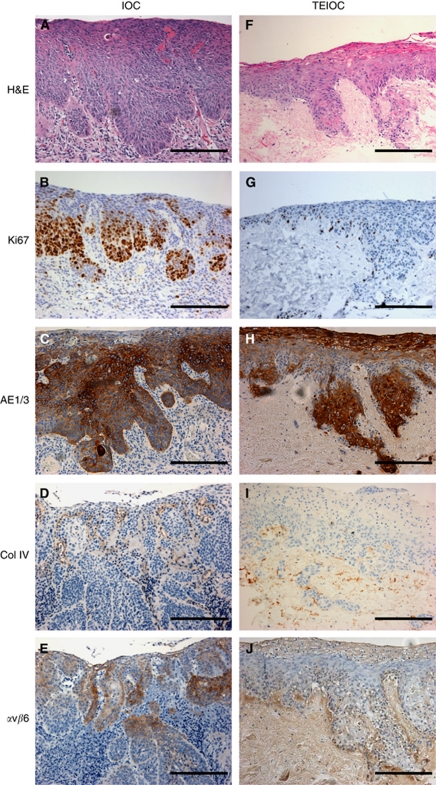
Representative sections of early invasive oral carcinoma (IOC) taken from patient biopsies (**A**–**E**) and a tissue-engineered invasive carcinoma (TEIOC) (**F**–**J**). TEIOC were generated by culturing OSCC (Cal27) and normal oral fibroblasts on DED at an air/liquid interface for 21 days. Sections have been stained with H&E (**A** and **F**) and immunohistochemically stained to show the pattern of expression of Ki67 (**B** and **G**), AE1/3 (**C** and **H**), collagen type IV (Col IV; **D** and **I**) and integrin *α*v*β*6 (**E** and **J**) and counterstained with haematoxylin. Scale bar indicates 200 *μ*m.

The progression from dysplasia to an invasive oral carcinoma is histopathologically defined when islands and cords of malignant epithelial cells arising from the overlying dysplastic mucosa invade through the basement membrane into the lamina propria. To model this, the TEDOM was further cultured for another 7 days, by which time histological analysis revealed that tumour cells had infiltrated the basement membrane and invaded the underlying stroma as evident by the presence of tumour islands within the connective tissue (Figure 6A and F). Similar results were observed when TEIOC models were made from the OSCC cell lines SCC9, SCC25, PE/CA-PJ34 and FaDu, and then cultured for 14–21 days at an air-to-liquid interface. In contrast, the OSCC cell line SCC4 failed to generate TEIOC models (Supplementary Figure 2). Immunohistochemical analysis confirmed that this TEIOC represents a histological picture of naturally occurring early invasive OSCC. Positive staining for pan-cytokeratin and cytokeratin 5/6 and negative staining for cytokeratin 7 and 20 confirmed that the invaded cells were of oral epithelial origin (Figure 6C and H, and data not shown). Collagen type IV staining showed that the basement membrane along the EDJ was completely disrupted and replicates the *in vivo* situation (Figure 6D and I). Although the epithelial cells in the invasive cancer model are not as proliferative as in the invasive OSCC, the TEIOC shows an abnormal proliferation profile with cells staining positively for ki67 throughout the epithelium and proliferating cells within the invading tumour island (Figure 6B and G). Expression of *α*v*β*6 was patchy but widely distributed throughout the dysplastic epithelium as well as in the islands of invasive OSCC, but expression was weak in the TEIOC model (Figure 6E and J) despite flow cytometric analysis showing strong *α*v*β*6 staining on monolayer cultures (Figure 5C).

## Discussion

In this study we present the development of a tissue-engineered 3D model of the oral mucosa using a native complex dermal matrix to produce three *in vitro* models demonstrating histopathological malignant transformation through dysplasia, carcinoma *in situ* to early invasive carcinoma. The models were closely compared with their *in vivo* counterpart with regard to the architectural and cytological changes observed. Furthermore, we used the models to investigate the expression of the integrin *α*v*β*6 at different stages of cancer progression and compared these with patient biopsies, as dysregulated expression of this integrin has been previously associated with dysplasia and carcinogenesis (Thomas *et al*, 2001b; Nystrom *et al*, 2006).

Histological examination of the TENOM revealed that normal keratinocytes proliferated to produce a stratified squamous epithelium several layers thick that is consistent with previously published reports (Cho *et al*, 2000; Bhargava *et al*, 2004; Moharamzadeh *et al*, 2008). To produce a model of oral dysplasia, NOKs were substituted with the dysplastic cell line, D20, or the malignant OSCC cell line, Cal27. The histological changes observed in both our TEDOM models are typical for a lesion classified as premalignant dysplasia with increased numbers of proliferating keratinocytes that are abnormally located in the supra-basal layers and a degree of atypical maturation and architecture.

Dysplastic keratinocytes derived from clinical lesions have an altered phenotype when cultured as 3D constructs with the degree of dysplasia not matching that of the original lesion (Gaballah *et al*, 2008). This may be because lesions are a heterogeneous mix of keratinocytes at different developmental stages and more atypical cells may possess a survival advantage in culture (Gaballah *et al*, 2008). The aim of the current study was to produce a model system that could easily be reproduced. The advantages of using a dysplastic cell line include reproducibility, standardisation and to avoid donor-dependant variations including age and site of isolation, which often arise when using donor-derived cells. Morphological and cytological changes are also consistent through serial passage for immortal cell lines, which is not the case for mortal dysplastic keratinocytes (Gaballah *et al*, 2008). Costea *et al* (2006) showed that DOK cells were invasive in collagen-based connective tissue organotypic models, whereas we found DOK cells to be noninvasive in our native DED-based models. The DOK cells are also nontumourigenic in mice (Chang *et al*, 1992), suggesting that their invasion capacity is restricted by native ECM but not by collagen alone and underscores the importance of using DED in models of dysplasia.

True to the malignant progression of OSCC, further culture of the Cal27 TEDOM led to breakdown of the basement membrane and extensive epithelial tumour cell invasion into the connective tissue component of the model that was analogous to early invasive OSCC *in vivo*. Stromal fibroblasts appear to be crucial for tumour invasion and progression (Kalluri and Zeisberg, 2006). During transition from normal epithelium to malignancy, some fibroblasts transdifferentiate into *α*-smooth muscle actin-expressing myofibroblasts that facilitate oral tumour cell invasion (Lim *et al*, 2011; Marsh *et al*, 2011). In this study marked invasion into the connective tissue of the TEIOC model was evident even in the presence of normal human oral fibroblasts (NOFs were *α*-smooth muscle actin negative by immunohistochemistry; data not shown). Using collagen-based models, Marsh *et al* (2011) showed increased invasion of OSCC cells into the ECM in the presence of myofibroblasts compared with NOFs, and no invasion was observed in models without fibroblasts (Marsh *et al*, 2011). This suggests that OSCC invasion into the DED of our models may be further enhanced if myofibroblasts were used in place of NOFs.

Immunohistochemical analysis has shown that expression of integrin *α*v*β*6 is altered during the transition from NOM though premalignant lesions to invasive oral cancer (Thomas *et al*, 1997, 2001b; Nystrom *et al*, 2006). It is also upregulated in mucosal development, wound healing and areca (betel) nut-mediated oral submucous fibrosis (Nystrom *et al*, 2006; Thomas *et al*, 2006; Moutasim *et al*, 2011). The *α*v*β*6 integrin appears to play an important role in oral cancer cell invasion by directly upregulating the expression of matrix metalloproteinase-9 and indirectly by activating TGF-*β* that then promotes myofibroblast formation (Thomas *et al*, 2001a; Lewis *et al*, 2004). The *α*v*β*6-dependent OSCC invasion also requires COX-2-dependent activation of Rac-1, via upregulation of PGE_2_ (Nystrom *et al*, 2006). Similar to the normal *in vivo* situation, we found *α*v*β*6 expression localised to the basal cells of the mucosal models even though flow cytometric analysis showed abundant cell surface expression on all epithelial cells before seeding onto the DED, showing that 3D culture alters the expression pattern of these molecules. Furthermore, similar *α*v*β*6 expression patterns were observed in both dysplastic and invasive models when compared with biopsy, providing evidence of the usefulness of these models in mimicking the situation *in vivo*. The dysplastic and invasive models may therefore be useful in future studies aimed at identifying the underlying mechanisms of *α*v*β*6-mediated tumour cell invasion.

One consideration, when producing the composites, was the ability to model the transformation from a dysplastic to an invasive phenotype. It was therefore important that the structural integrity of the 3D construct was maintained throughout the culture period. Bovine or rat-based collagen is often used for 3D organotypic models because of their ease of use and reproducibility, but these lack the complex structural heterogeneity and degree of collagen fibre crosslinking that is present in mature human oral connective tissue and they are not biostable over long culture periods with artificial invasion possible (Wolf *et al*, 2009). Furthermore, we and others have shown that these models do not contain a well-defined, continuous basement membrane between the epithelium and the connective tissue that is crucial in the morphological development of the epithelium and its attachment to the connective tissue (Chung *et al*, 1997; Ralston *et al*, 1999; Yadev *et al*, 2011) as well as in defining the boundary of tumour invasion. The collagen composition, organisation, fibre size and orientation that produce heterogeneous pore sizes within the DED are similar to what is seen *in vivo* (Wolf *et al*, 2009). Therefore, the use of DED more closely models the connective tissue element of the oral mucosa compared with collagen gels and enables long-term culture of the models for up to 28 days. We suggest that the complex nature of DED is the most physiologically relevant material to test the ability of tumour cells to invade into the connective tissue *in vitro*. However, these models do not contain a vasculature or immune cell component that may influence tumour cell invasion and hence may not be truly representative of *in vivo* invasion.

In summary, we have established a novel 3D model of oral dysplasia, carcinoma *in situ* and early invasive carcinoma that allows the study of architectural and cytological changes within a multilayered construct in a controlled microenvironment. The use of cell lines and commercially available DED ensures that the model is reproducible. Further applications could involve use in final-stage anticancer drug screenings or adaptation of this model to study other diseases and systems.

## Figures and Tables

**Table 1:** Antibodies and antigen retrieval conditions used in this study

**Marker**	**Antibody clone**	**Dilution/final concentration**	**Antigen retrieval**	**Source**
Ki67	MIB-1	1 : 50	0.01 M sodium citrate buffer	Dako, Copenhagen, Denmark
AE1/AE3	AE1/AE3	1 : 100	0.01 M sodium citrate buffer	Dako
Collagen IV	COL-94	1 : 500	0.01 M sodium citrate buffer	Sigma, Poole, UK
*α*v*β*6	6.2A1	0.5 *μ*g ml^–1^	Proteinase K	Generated as described in Weinreb *et al* (2004)
