# Supplementary material for: Development of tissue-engineered models of oral dysplasia and early invasive oral squamous cell carcinoma
Source: Br J Cancer. 2011 Oct 11;105(10):1582–92. doi: 10.1038/bjc.2011.403 (PMC3242522; doi:10.1038/bjc.2011.403)
Supplement: Supplementary Figure 1 [file bjc2011403x1.ppt]

## Slide 1
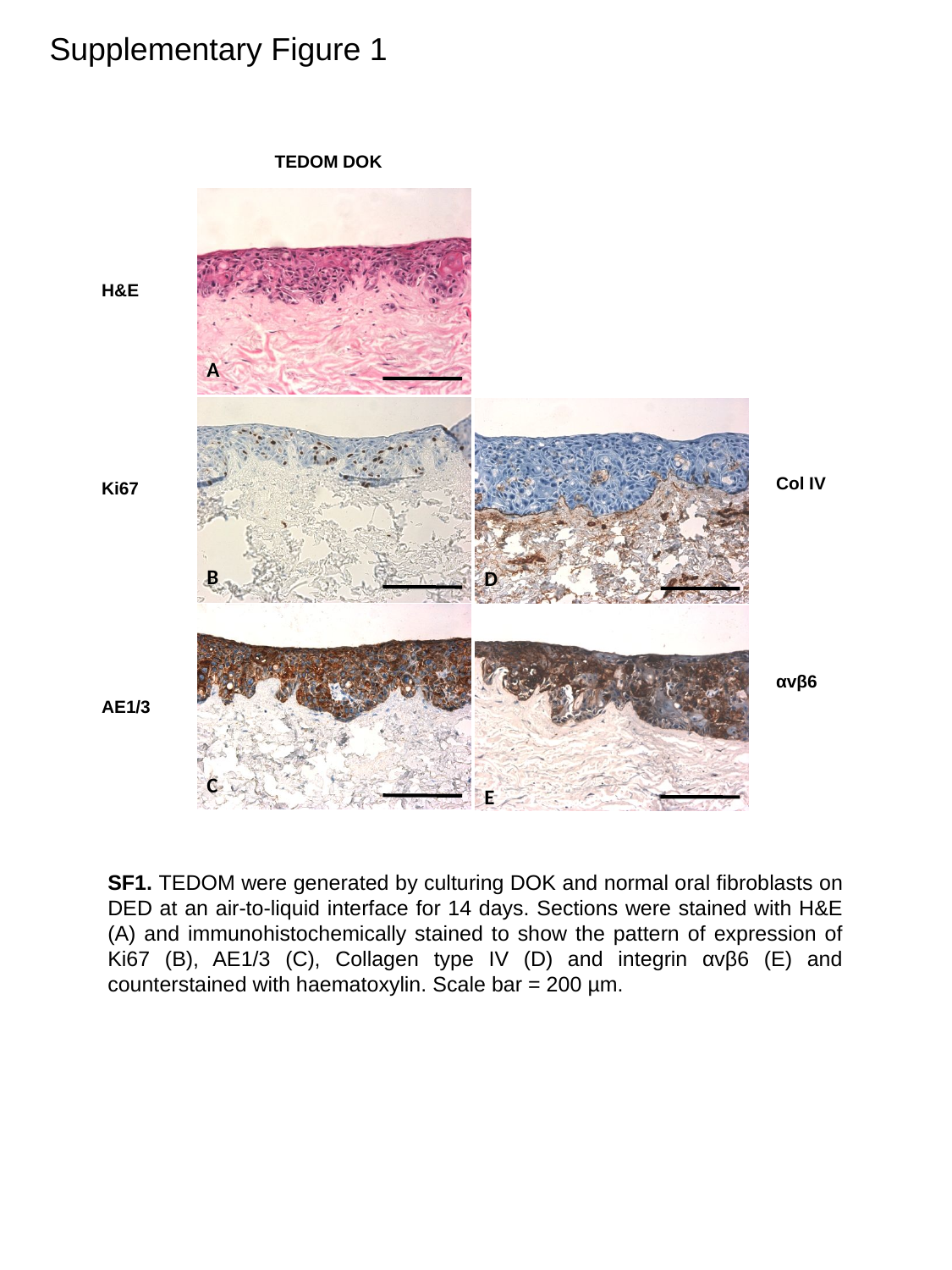

Supplementary Figure 1
TEDOM DOK
H&E
A
D
E
Col IV
αvβ6
Ki67
B
AE1/3
C
SF1. TEDOM were generated by culturing DOK and normal oral fibroblasts on DED at an air-to-liquid interface for 14 days. Sections were stained with H&E (A) and immunohistochemically stained to show the pattern of expression of Ki67 (B), AE1/3 (C), Collagen type IV (D) and integrin αvβ6 (E) and counterstained with haematoxylin. Scale bar = 200 µm.
