# Supplementary material for: Development of tissue-engineered models of oral dysplasia and early invasive oral squamous cell carcinoma
Source: Br J Cancer. 2011 Oct 11;105(10):1582–92. doi: 10.1038/bjc.2011.403 (PMC3242522; doi:10.1038/bjc.2011.403)
Supplement: Supplementary Figure 2 [file bjc2011403x2.ppt]

## Slide 1
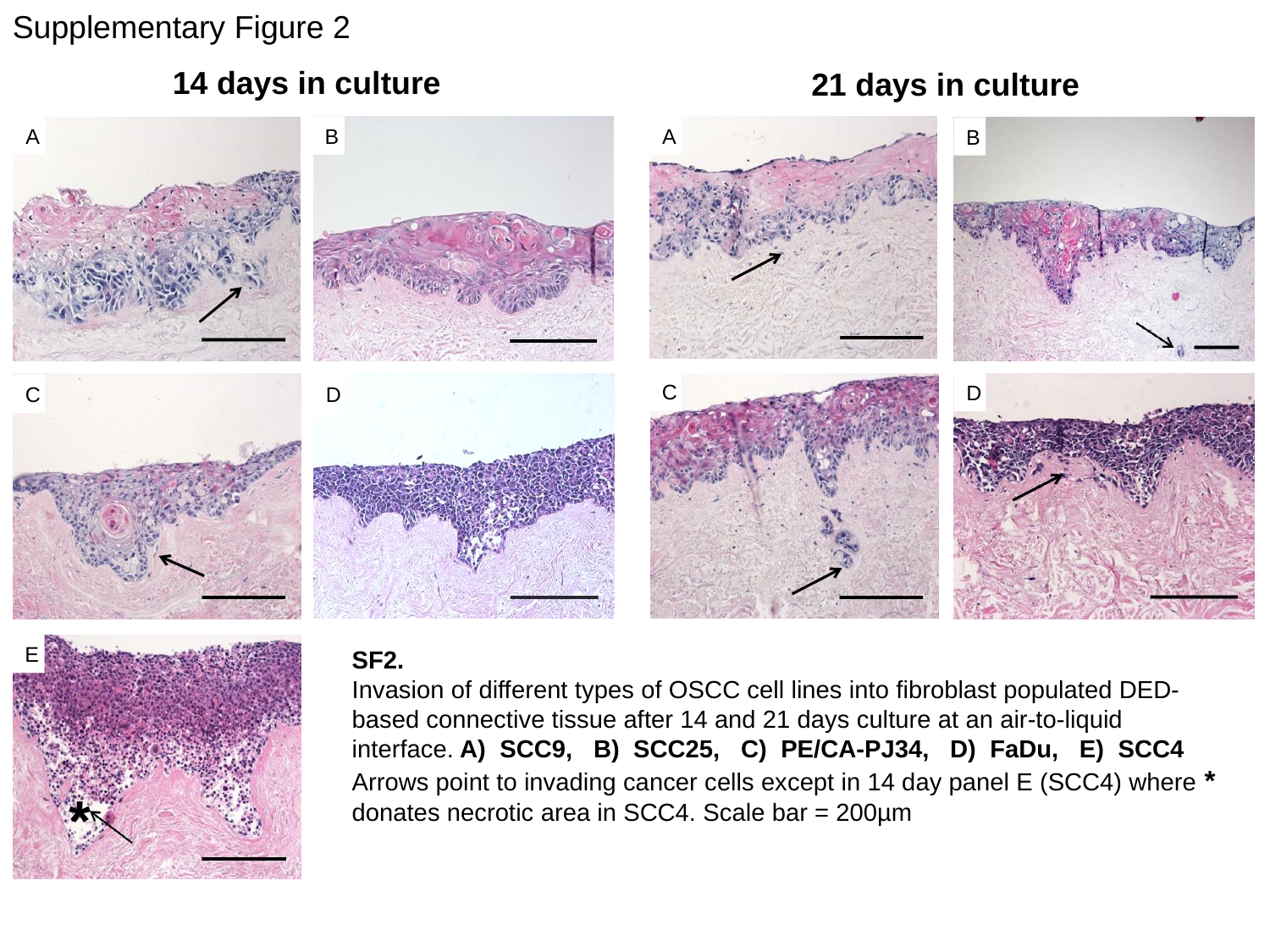

Supplementary Figure 2
14 days in culture
21 days in culture
A
B
C
D
A
B
C
D
E
SF2.
Invasion of different types of OSCC cell lines into fibroblast populated DED-based connective tissue after 14 and 21 days culture at an air-to-liquid interface. A) SCC9, B) SCC25, C) PE/CA-PJ34, D) FaDu, E) SCC4
Arrows point to invading cancer cells except in 14 day panel E (SCC4) where * donates necrotic area in SCC4. Scale bar = 200µm
*
